# Is the difference in neonatal blood glucose concentration of caesarian and vaginally delivered term infants requiring separated reference intervals?

**DOI:** 10.1186/1756-0500-5-519

**Published:** 2012-09-24

**Authors:** Mulugeta Melkie, Mahilet Yigeremu, Paulos Nigussie, Tilahun Teka, Samuel Kinde

**Affiliations:** 1 Department of Medical Laboratory Science, Arbaminch University, Arbaminch, Ethiopia; 2 Faculty of Medicine, Addis Ababa University, Addis Ababa, Ethiopia; 3 Ethiopian Health and Nutrition Research Institute, Addis Ababa, Ethiopia

**Keywords:** Reference interval, Neonatal hypoglycemia, Vaginal delivery, Cesarean section, Cord blood glucose, Robust method

## Abstract

**Background:**

Mode of delivery has been reported to affect the cord blood glucose level of newborns. Vaginally delivered (VD) newborns were found to have relatively increased concentration of cord blood glucose than those delivered by cesarean section (CS). The aim of this study is thus to determine whether the difference in cord blood glucose concentration among VD and CS newborns is necessitating partitioned reference intervals (RIs) for the laboratory diagnosis of neonatal hypoglycemia.

**Methods:**

A total of 60 newborns were included from Tikur Anbessa Specialized Hospital (TASH). Cord blood samples were collected and analyzed for glucose by Glucose-oxidase (GOD-PAP) method in TASH laboratory using HumaStar 300 from November 2010 to April 2011. All pre-analytical, analytical and post-analytical aspects were thoroughly controlled. A robust method was used for the determination of reference intervals using MedCalc® software Version 11.6.1.

**Results:**

VD newborns accounted for 71.7% (n = 43) while the CS newborns accounted for 28.3% (n = 17). No statistically significant difference was noted in the studied demographic variables among VD and CS newborns except for blood glucose level. The RIs were then determined to be 2.46-6.85 mmol/l and 2.46-5.04 mmol/l for VD and CS newborns respectively. The combined RI was 2.24-6.48 mmol/l.

**Conclusion:**

Combined RI better be used for the interpretation of cord blood glucose values in VD and CS newborns. Cord blood glucose concentrations of 2.24 mmol/l can be used as statistical estimates of cut off points for neonatal hypoglycemia in newborns irrespective of their mode of deliveries.

## Background

Labor is a stress both to the mother and the fetus. During labor the presence of pain, fatigue, physiologic alterations and maternal anxiety stresses the mother subsequently increasing maternal catecholamine [1,2]. Fetal catecholamine production also increases up to ten fold compared to pre-labor levels as a result of the stress [1].

Both animal and human studies have showed that catecholamine release has profound difference in different modes of deliveries. Particularly, the production of catecholamines is higher during normal vaginal delivery (VD) than during cesarean section (CS) which can be explained by the increased stress in VD [3,4]. This labor induced fetal catecholamine surge is presumed to provide the neonate with an important mechanism of adaptation to the extra-uterine environment [5]. Besides, the catecholamines made newborns delivered through VD to acquire an increased level of blood glucose than their counterparts delivered through elective CS [6]. This may be resulted from hyperglycemic effect of the increased catecholamine release as it appears to promote glycogenolysis [7]. Although there are no evidences, the reduced glucose level in CS newborns might slightly be confounded by acquisition of low glucose level prior delivery by the fetus as it is receiving its entire supply of glucose from the maternal circulation by facilitated diffusion via the placenta in a continuous manner [8].

The aim of this pilot study is then to determine whether separated reference intervals (RIs) are required for the interpretation of neonatal blood glucose levels in VD newborns and CS newborns. We studied the null hypothesis that the RI is not the same for blood glucose levels in VD and CS newborns.

## Methods

A cross sectional study was conducted in Obstetrics and Gynecology (OBGY) department of Tikur Anbessa Specialized Hospital (TASH) from November 2010 to April 2011. Newborns with over 37 weeks of gestation, ≥2500 g birth weight and no history of fetal problems were sequentially enrolled in the study and their cord blood was collected. Preterm newborns, newborns with <2500 g birth weight, and newborns requiring intensive resuscitation and care, newborns from mothers with documented ante- and/or intra-partum complications (gestational diabetes, HIV, hepatitis B/C, eclampsia) were excluded.

The proposal of this study was reviewed by the Department of Medical Laboratory Sciences research review committee and by the Institutional Review Board (IRB) of Addis Ababa University, Faculty of Medicine. Informed (written) consent was obtained from mothers before delivery.

The pre-analytical, analytical and post-analytical phases were controlled throughout the study. Moreover, a pilot study was conducted before the actual data collection. Demographic data were collected using pretested questionnaire. HumaStar 300 (human diagnostics worldwide, Germany) was used for laboratory analyses. Glucose was determined by the Glucose-oxidase (GOD-PAP) method supplied by HUMAN®.

The data obtained from the analyzer and the questionnaire were entered in Microsoft excel sheet and analyzed by MedCalc® software Version 11.6.1. Comparison of blood glucose concentration and selected demographic variables between VD newborns and CS newborns were done using independent sample t-test and Chi-square test. Tukey test and D’Agostino-Pearson (DAP) test were used to detect outlier glucose values and to determine normality of distributions of glucose values respectively. Then, the upper and lower end points covering 95% of the reference value of glucose were determined with their respective 90% confidence intervals (CIs) by using the robust method, according to the IFCC/CLSI recommendation [9]. Haris and Boyd rule was applied to determine whether partitioning of RIs should be done.

## Results

### Analytical performance of the GOD-PAP method

Intra-assay coefficients of variations (CVs) were 0.72% and 1.41% for normal and pathological control sera respectively. Similarly, the inter-assay CVs were 1.29% and 3.05% for normal and pathological control sera respectively. Commercial quality control sera (HUMATROL N and P) were included in every session of analyses. LJ charts were then plotted and all the quality control results were in the acceptable limits.

### Demographic data of study participants

Sixty (60) study participants were included in this study among which 28 (46.7%) were females and 32 (53.3%) were males. VD newborns accounted for 71.7% (n = 43) while the CS newborns accounted for 28.3% (n = 17). The mean height, weight and head circumference at birth were 50.9 cm, 3120 g and 35.25 cm respectively. 39 (65%) of the mothers were primiparous whereas 21 (35%) were multiparous.

### RI determination

**Figure 1:**
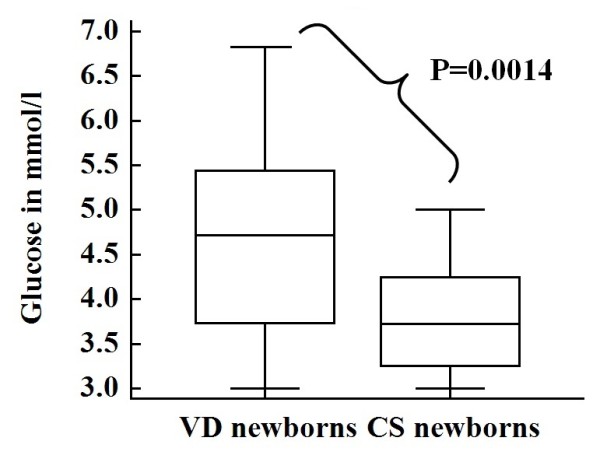
Box and Whisker plot that shows the difference among VD and CS newborns in cord blood glucose levels.

The minimum and maximum concentrations of glucose in VD newborns were 3.0 mmol/l and 6.83 mmol/l respectively with mean value of 4.71 mmol/l. Likewise, 3.0 mmol/l and 5.0 mmol/l were the minimum and maximum concentrations of glucose in CS newborns while the mean value was 3.78 mmol/l (Tables 1, 2 and Figure 1).

**Table 1:** Comparison of blood glucose and selected demographic variables between VD and CS newborns

	**Vaginal delivery (n = 43)**	**Cesarean section (n = 17)**	***p*-value**
Gestational weeks	Mean = 38.4	Mean = 38.9	0.1841^*^
	SD = 1.11	SD = 1.76	
Weight (g)	Mean = 3066.3	Mean = 3255.9	0.1086^*^
	SD = 335.7	SD = 549.4	
Height (cm)	Mean = 50.84	Mean = 51.06	0.8037^*^
	SD = 3.28	SD = 2.56	
Head circumference (cm)	Mean = 35.05	Mean = 35.77	0.4151^*^
	SD = 3.48	SD = 1.39	
Cord blood Glucose (mmol/l)	Mean = 4.71	Mean = 3.78	0.0014^*^
	SD = 1.07	SD = 0.59	
Sex			0.8035^†^
Parity			0.7411^†^

* Independent sample t-test.

† Chi-square test.

**Table 2:** Summary of RI determinations of glucose (mmol/l) in VD and CS newborns

	**n**	**Outlier (Tukey)**	**Min. value**	**Max. value**	**Mean (95% CI)**	**SD**	**DAP test**	**Lower limit (90% CI)**	**Upper limit (90% CI)**	**RI**	**Harris and Boyd**
**VD newborns**	43	No	3.0	6.83	4.71	1.07	P = 0.303	2.46	6.85	2.46-6.85	**Separated RIs should be used**
					(4.37-5.03)			(2.10-2.90)	(6.37-7.31)		
**CS newborns**	17	No	3.0	5.0	3.78	0.59	P = 0.552	2.46	5.04	2.46-5.04	
					(3.48-4.08)			(2.07-2.82)	(4.51-5.47)		
**Combined**	60	No	3.0	6.83	4.44	1.04	P = 0.116	2.24	6.48	2.24-6.48	
					(4.17-4.71)			(1.90-2.57)	(6.02-6.89)		

## Discussion

In this study, we compared the blood glucose concentration, gestational weeks, weight, height and head circumference at birth, and sex and parity distributions among VD newborns and CS newborns (Table 1). We found no statistically significant difference in all aforementioned demographic variables among newborns delivered through VD and CS. However, the cord blood glucose level was significantly higher in VD newborns than CS newborns (Figure 1). A similar finding has been reported by Marom and his colleagues in which neonatal blood glucose concentration was higher in VD newborns than CS newborns [6].

Our special interest in this study was to determine whether the neonatal blood glucose difference among these groups is necessitating partitioned RIs. As clearly depicted in Table 2, there were no outlier glucose values detected in both groups. At the same time, the distributions were Gaussian/normal in both groups. The RIs were then determined to be 2.46-6.85 mmol/l and 2.46-5.04 mmol/l for newborns delivered through VD and CS respectively. Harris and Boyd analysis suggested that partitioning should be done as the two RIs are far apart from each other. However, the lower limit of both RIs is almost identical. As a result, partitioning the reference intervals may not be relevant clinically specially in the diagnosis and management of neonatal hypoglycemia. Even though we failed to determine the glucose concentrations in the subsequent hours after delivery, it has been described in literatures that the difference in glucose level might not be reflected in subsequent hours (≥ 2 hours) after delivery as a result of larger glucose decrease in VD newborns from the action of residual insulin secretion and slight glucose increase in CS newborns [6,10]. Hence, it might be important to apply the combined RI (2.24-6.48 mmol/l) for the interpretation of glucose results of infants older than two hours irrespective of the mode of delivery.

Since we have used a robust method for determinations of the RIs, the lower limits were in close proximity to 2SDs lower than the respective mean values. Hence, the lower limits can be used as statistical tools to define neonatal hypoglycemia that are associated with clinical signs leading to neuro-developmental squeal [11]. Therefore, it is possible to statistically estimate that newborns with cord blood glucose level of lower than 2.24 mmol/l, irrespective of mode of deliveries, could be considered as on greater risk of developing symptomatic hypoglycemia that might be accompanied with clinical presentations.

## Conclusion

From this study, we conclude that combined RIs better be used for the interpretation of cord blood glucose values for VD and CS newborns. Cord blood glucose concentrations of 2.24 mmol/l can be used as statistical estimates of cut off points for neonatal hypoglycemia in newborns irrespective of mode of deliveries.

## Competing interests

The authors declared no competing interests in this research. In fact, the research was financially supported by Addis Ababa University; and other non-financial supports were also obtained from Mesroy international plc (Reagents) and Medcalc Software Company (statistical software).

## Authors’ contributions

MM, TT and SK have participated in the conception and design of the study. MY and TT have participated in the selection of study participants. MM and PN have participated in the laboratory analysis and acquisition of data. All authors have participated in preparing and critically reviewing the draft manuscript. All authors also have read and approved the final manuscript.
